# Optimizing PCL/PLGA Scaffold Biocompatibility Using Gelatin from Bovine, Porcine, and Fish Origin

**DOI:** 10.3390/gels9110900

**Published:** 2023-11-14

**Authors:** Mina Ghafouri Azar, Lucie Wiesnerova, Jana Dvorakova, Petra Chocholata, Omid Moztarzadeh, Jiri Dejmek, Vaclav Babuska

**Affiliations:** 1 Department of Medical Chemistry and Biochemistry, Faculty of Medicine in Pilsen, Charles University, alej Svobody 76, 323 00 Pilsen, Czech Republic; mina.ghafouri@lfp.cuni.cz (M.G.A.); lucie.wiesnerova@lfp.cuni.cz (L.W.); jana.dvorakova@lfp.cuni.cz (J.D.); petra.chocholata@lfp.cuni.cz (P.C.); 2 Department of Stomatology, University Hospital Pilsen, Faculty of Medicine in Pilsen, Charles University, alej Svobody 80, 304 60 Pilsen, Czech Republic; omid.moztarzadeh@lfp.cuni.cz; 3 Department of Biophysics, Faculty of Medicine in Pilsen, Charles University, alej Svobody 76, 323 00 Pilsen, Czech Republic; jiri.dejmek@lfp.cuni.cz

**Keywords:** polycaprolactone, gelatin, tissue engineering, biocompatibility, cell viability

## Abstract

This research introduces a novel approach by incorporating various types of gelatins, including bovine, porcine, and fish skin, into polycaprolactone and poly (lactic-co-glycolic acid) using a solvent casting method. The films are evaluated for morphology, mechanical properties, thermal stability, biodegradability, hemocompatibility, cell adhesion, proliferation, and cytotoxicity. The results show that the incorporation of gelatins into the films alters their mechanical properties, with a decrease in tensile strength but an increase in elongation at break. This indicates that the films become more flexible with the addition of gelatin. Gelatin incorporation has a limited effect on the thermal stability of the films. The composites with the gelatin show higher biodegradability with the highest weight loss in the case of fish gelatin. The films exhibit high hemocompatibility with minimal hemolysis observed. The gelatin has a dynamic effect on cell behavior and promotes long-term cell proliferation. In addition, all composite films reveal exceptionally low levels of cytotoxicity. The combination of the evaluated parameters shows the appropriate level of biocompatibility for gelatin-based samples. These findings provide valuable insights for future studies involving gelatin incorporation in tissue engineering applications.

## 1. Introduction

With the progress of science and technology, researchers have developed various synthetic polymers or their derivatives to create new composite scaffolds or films that can replace damaged functional tissues or organs [1,2,3,4,5]. A significant challenge remains in the development of biomedical films that effectively support cell adhesion and proliferation and provide the necessary mechanical strength to maintain the flexibility of internal tissues without rupturing during procedures. However, the engineering of biocomposites or blends is challenging due to the need to meet specific criteria, such as biocompatibility, non-allergenicity, non-toxicity, non-carcinogenicity, sterilizability, good antibacterial, and favorable mechanical properties while retaining their original characteristics [6,7,8]. An essential consideration in the design of these biofilms is the physicochemical properties of the primary components and their degradation behavior. These factors directly influence the mechanical properties of the resulting films.

Recent reports have highlighted the use of biodegradable polycaprolactone (PCL) as a major component in the synthesis of biological films, particularly for tissue engineering [9,10,11] and long-term drug delivery systems [12,13,14]. PCL, which is approved by the US Food and Drug Administration for biomedical research, is favored due to its biocompatibility, high water resistance, viscoelasticity, and rheological properties [15,16]. The excellent characteristics of PCL and its blending with polysaccharides, proteins or synthetic polymers have been utilized in various ways, such as PCL/chitosan [17,18], PCL/starch [19], chitosan/PCL/polypyrrole [20], PCL/PVA/silk fibroin composites [21], among others. Several studies have documented suitable properties of biomaterials based on the combination of PCL, gelatin, and chitosan. For example, Zhang et al. reported optimal porosity, pore size, and mechanical stability, as well as suitable swelling and hydrophilicity. The scaffold exhibited excellent biocompatibility, induced high collagen secretion, accelerated blood clotting, and acted as a barrier against external cell invasion [22]. The same composition of scaffold with fish gelatin was evaluated by Gomes et al. They found excellent dimensional stability, optimal physical properties, and higher cell adhesion capabilities, suggesting the potential for cell-based therapies in skin regeneration [23]. Poly (lactic-co-glycolic acid) (PLGA) is a bioresorbable polymer widely used in the fields of drug delivery and tissue engineering. Its exceptional biocompatibility is attributed to the degradation process of its individual monomeric units, lactic acid, and glycolic acid. The ester linkages in PLGA undergo hydrolysis during degradation. Lactic acid is metabolized through various enzymatic pathways and is eliminated as carbon dioxide and water. Glycolic acid, on the other hand, is primarily excreted through urine after undergoing oxidative metabolism in the liver [24,25]. In a study by Ouyang et al., PLGA was utilized for the cultivation of bone marrow stromal cells [26]. Lu et al. designed microfabricated PLGA particles for pulsatile release of a stimulator of interferon gene (STING) agonist, aiming to enhance cancer therapy [27]. More recently, Koerner et al. incorporated Riboxxim, along with an antigen, into PLGA particles for cancer immunotherapy. This PLGA particle vaccine demonstrated notable outcomes, including retardation of primary tumor growth, prevention of metastases, and prolonged survival in preclinical tumor models [28,29].

The combination of PCL and PLGA polymers offers synergistic properties and a wide range of applications due to their complementary characteristics [30]. This combination allows for tailoring the properties of the resulting composite materials by leveraging the strengths of each polymer. For example, PCL can improve the mechanical properties and processability of PLGA, while PLGA can enhance the degradation and drug release behavior of PCL. The PCL/PLGA combination has found applications in scaffold fabrication, drug delivery systems, and tissue engineering constructs [31]. These hybrid systems have been explored for various purposes, including bone tissue engineering, cartilage regeneration, wound healing, and controlled release of therapeutics [32]. On the other hand, the combination of PCL and PLGA materials has limitations in terms of their resistance to stretching forces and potential adverse effects. To address these issues and improve their application in tissue engineering, new materials are urgently needed. One promising approach is to add a third component to the composite. By incorporating gelatin, the elasticity of the membrane is improved, and there is a significant enhancement in cell adhesion, infiltration, tissue integration, and regeneration [33].

Gelatin is a protein derived from collagen, the main component of the extracellular matrix in animal connective tissues. Gelatin composition and properties can vary depending on the source [34]. The amino acid composition is a primary difference among gelatins from different sources. Bovine gelatin, for example, has a higher content of proline and hydroxyproline, resulting in higher gel strength and gelling temperature, and forming firmer and more stable gels compared to fish gelatin. Porcine gelatin is similar to bovine gelatin in terms of amino acid composition but may have slight variations [35]. Fish gelatin, however, has a lower proline and hydroxyproline content and usually contains more glycine and alanine. This leads to lower gel strength, making it suitable for applications where a softer gel or film is desired, such as wound dressings [36].

The combination of PCL, PLGA, and peptides or proteins has been extensively studied and utilized in various biomedical applications [37,38]. Tricomponent systems combining PCL, PLGA, and gelatin offer tailored properties for tissue engineering and drug delivery. The composite membrane exhibits synergistic effects, enhancing mechanical strength and biocompatibility. It combines the flexibility and stability of PCL, controlled degradation of PLGA, and the cell-interaction properties of gelatin. Applications include scaffolds for bone, cartilage, and skin regeneration, as well as controlled drug release systems. The composition and ratio of components can be adjusted for the desired mechanical properties, degradation rates, surface tribological performance, and bioactivity [39].

In our study, we fabricated biodegradable composite films by combining PCL/PLGA with gelatin from different sources, including bovine, porcine, and fish. Currently, there is a lack of comprehensive research on the development of composite films using PCL and PLGA in conjunction with various types of gelatins for targeted biomedical applications in tissue engineering. Our hypothesis is that blending bioresorbable polymers with gelatin will not only promote excellent biocompatibility but also create porous structures that facilitate cell ingrowth and expedite the healing process.

## 2. Results and Discussion

### 2.1. Morphology

**Figure 1:**
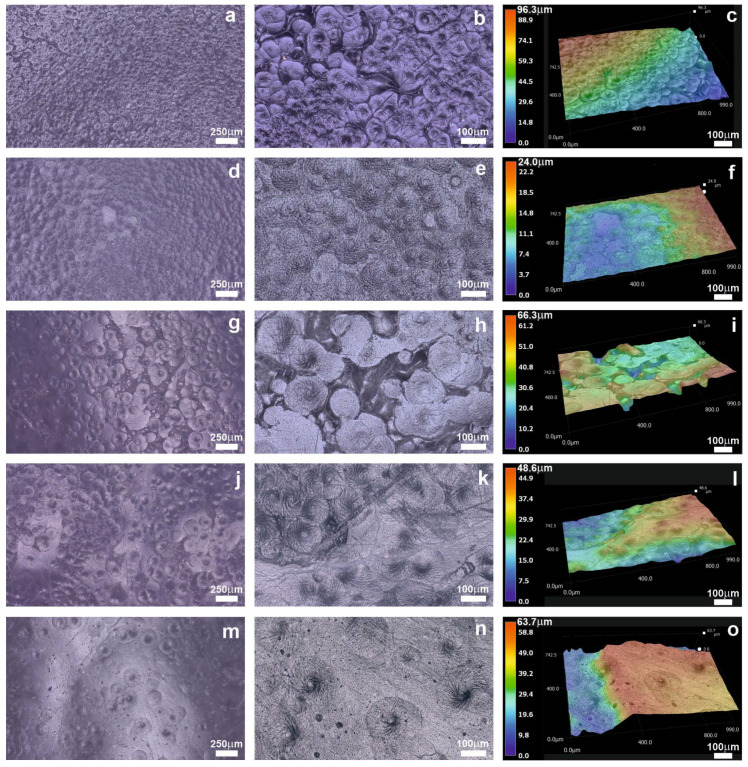
Morphology of different composites in 2D and 3D. The first column scale bar is 250 μm, the second and third column scale bar is 100 μm. The composites are labeled as follows: (**a**–**c**) PC; (**d**–**f**) PCG; (**g**–**i**) PCGB; (**j**–**l**) PCGP; and (**m**–**o**) PCGF.

The surface features of various composite scaffolds were investigated and compared using a digital microscope at magnifications 100x and 300x; a 3D version was also taken at 300x (Figure 1). 

Initial observations of the PC sample (Figure 1a–c) depict the presence of numerous small circular structures on the surface, attributed to the crystallinity of the PCL polymer. In the PCG composite, where PLGA is added to the PCL matrix (Figure 1d–f), the surface undergoes noticeable changes and becomes smoother, due to the increased degradability of the composite [40].

The images revealed distinct features especially when the gelatins were incorporated into the composites. The addition of gelatin from bovine skin (Figure 1g–i) resulted in the formation of larger circular domains on the composite surface, indicating the significant influence of the bovine gelatin’s molecular characteristics and properties on the composite’s morphological changes. Similarly, the incorporation of porcine gelatin (Figure 1j–l) also led to the presence of circular structures, although to a lesser extent when compared to the bovine gelatin. Conversely, the composite containing the fish gelatin (Figure 1m–o) exhibited a lower occurrence of circular domains. The variations in surface morphology can be attributed to the different kinds of gelatin used, including their structural and molecular characteristics as well as their influence on degradation and crystallinity [41]. Previous studies have established that pure PCL film possesses a smooth surface devoid of any holes. However, the inclusion of PLGA or gelatins into PCL films leads to the formation of numerous circles and holes throughout the surface, accompanied by considerable surface collapse [9].

### 2.2. Mechanical Properties

**Figure 2:**
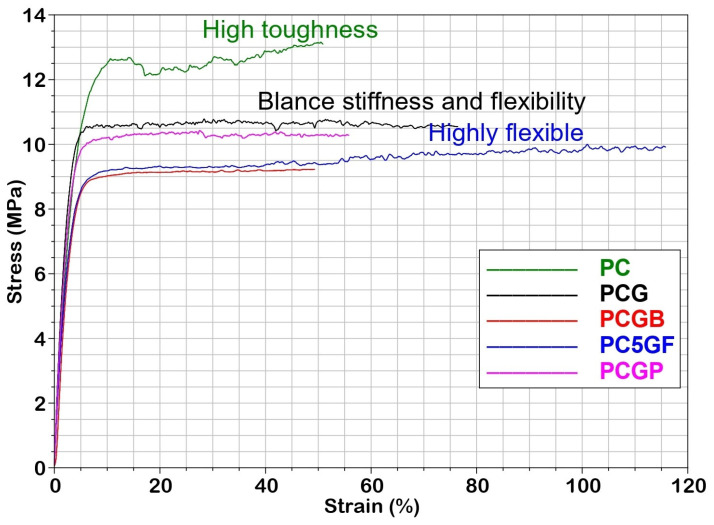
Tensile stress-strain curves for various types of composite films at room temperature.

The tensile stress-strain curves of all the samples are shown in Figure 2. The results indicate that the incorporation of gelatin has altered the tensile properties compared to the matrix. For instance, the PCL matrix (PC) exhibited an ultimate strength of 13.22 MPa with an elongation at break of 52%. Upon incorporating PLGA into PC (PCG), the strain percentage increased significantly compared to PC, reaching 75.98%. However, it is noteworthy that the stress experienced a decrease, measuring 10.55 MPa. However, the gelatin-containing samples displayed lower ultimate strength, ranging from 9.19 to 10.55 MPa, but interestingly, they showed different elongation at break ranging from 48.12% to 115.8%. Consequently, both the tensile strength and strain at break, and thus the tensile toughness (represented by the area under the stress-strain curve), were significantly affected by the incorporation of PLGA or gelatins.

The composite film with fish gelatin (PCGF) exhibited the highest flexibility with a strain at break of 117%. Additionally, the initial stiffness (as observed in the initial stress-strain curve segment) slightly decreased for all composites compared to the PC sample. This reduction may be attributed to the decreased crystallinity observed in our digital microscopy results. The PCGF sample exhibited a comparable modulus to composites with bovine and porcine gelatins [42]. However, the films with mammalian gelatins displayed lower in elongation values. These differences in mechanical properties may be attributed to the varying levels of renaturation achieved during the film forming process. Previous studies have demonstrated that porcine gelatin films with higher renaturation levels exhibit higher stress at break when compared to fish gelatin [43]. The presence of PLGA or gelatins creates a physical barrier between the PCL molecules, preventing them from forming strong crystalline structures. The disruption of chain mobility also inhibits the formation of strong bonds necessary for toughness. The limited miscibility of the two polymers further reduces intermolecular forces, thereby decreasing the material’s toughness. The tensile strength of these blend films is sufficient, making them suitable for tissue engineering purposes. 

### 2.3. Thermal Properties

**Figure 3:**
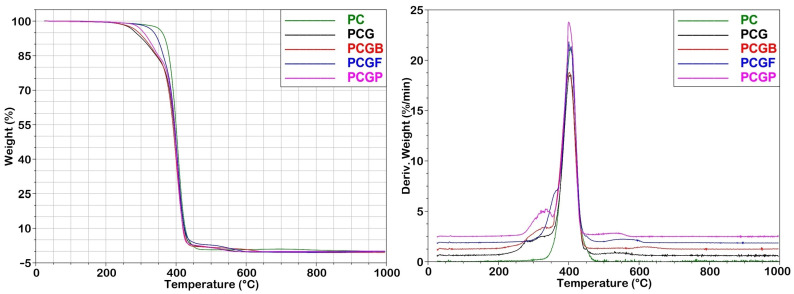
Thermogravimetric analysis (TGA) and derivative thermogravimetric (DTG) curves of different types of composite films performed on samples from 30 to 800 °C at a ramp rate of 10 °C/min under a nitrogen atmosphere.

Thermal stability is a critical parameter that must be evaluated to determine the degradation behavior of composite films, as the degradation process can produce small molecules or byproducts that may affect the chemical composition of the polymer and its biocompatibility. In Figure 3, the TGA and DTG graphs of different types of composite films are displayed.

The TGA curve of the basic PC sample exhibits a single-stage degradation pattern with an initial decomposition temperature (IDT) of approximately 350 °C. This degradation occurs immediately at a temperature of 100 °C, resulting from the breakdown of the polyester chains through pyrolysis [44]. The PCG films show a two-stage degradation pattern. The first stage occurs in the range from 244 to 349 °C, followed by the second stage between 349 and 464 °C, with negligible changes observed above 464 °C. The initial degradation step can be attributed to random chain scission of the polyesters, while the second stage involves more specific chain scission of the main chain [45]. Incorporating gelatins into the composite films does not significantly alter the thermal degradation pattern observed for the individual polymers (PCL and PLGA), although there is a slight shift of the primary curve towards higher temperatures. All the composites exhibit lower decomposition temperatures compared to pure PCL. The thermal stability of the PC sample is decreased by adding of PLGA (PCG sample). This trend can be reversed by the addition of gelatins, the best type appears to be fish gelatin with decomposition beginning at 320 °C. This phenomenon is likely due to the gelatin chains, which remain random in solution and interact more intensely as water evaporates from the sample [46]. Nevertheless, complete decomposition of all the composites occurs at ~465 °C. The TGA curves indicate that all the compositions are thermally stable and can withstand temperatures of at least 245 °C, making these films suitable for various biomedical applications.

### 2.4. Biodegradability Test

The biodegradability of the composite films was evaluated by immersing scaffolds in a simulated body fluid (SBF) at 37 °C for 24 days. The list of all the results is provided in Table 1, which shows the change in weight loss of the composite films over time. In general, the weight of all the composite films decreased after 24 days. According to the results, all the samples are biodegradable and their weight loss is in the range of 11.70% and 28.40%. The PC and PCG samples without gelatin incorporation have the lowest biodegradability, 11.70% and 15.15%, respectively.

PCL is commonly used in tissue engineering scaffolds due to its well-established biocompatibility. Although, its inherent hydrophobicity and crystallinity make it resistant to rapid degradation in vivo, necessitating modifications to enhance its biocompatibility. In order to enhance PCL’s degradability, gelatin is a suitable, naturally occurring, biodegradable, and biocompatible material. After incorporating different types of gelatins, the biodegradability increased in the PCGB, PCGP, and PCGF to 16.22%, 21.50%, and 28.40%, respectively. Also, our results support the findings that the addition of gelatins in a PCL/PLGA matrix increases the biodegradability of the composite materials. The degree and rate of degradation of the gelatin from different sources may be influenced by the type of proteases at the site of the biomaterial application in vivo as these enzymes cleave gelatin at specific sites, according to the primary amino acid sequence. This different degree of degradation can thus affect the long-term stability of the biomaterial [47].

### 2.5. Hemolytic Test

The hemocompatibility test measures the compatibility of the material with blood cells, specifically the extent of hemolysis, which is the breakdown of red blood cells. As shown in Table 1, all the samples exhibited hemolysis values of less than 5%, indicating a high level of hemocompatibility. The results indicate that all the samples are highly hemocompatible with hemolysis percentages ranging from 4.2% to 4.7%. Based on the results presented in the study by Ajmal et al. [48], it can be inferred that the incorporation of gelatins in the PCL/PLGA composite did not result in a significant alteration in its hemocompatibility. Therefore, the composite material, including the PCL/PLGA component, maintains high hemocompatibility, indicating favorable inherent properties that remain nearly unchanged even with the addition of gelatin.

### 2.6. Cell Adhesion and Proliferation Test

**Figure 4:**
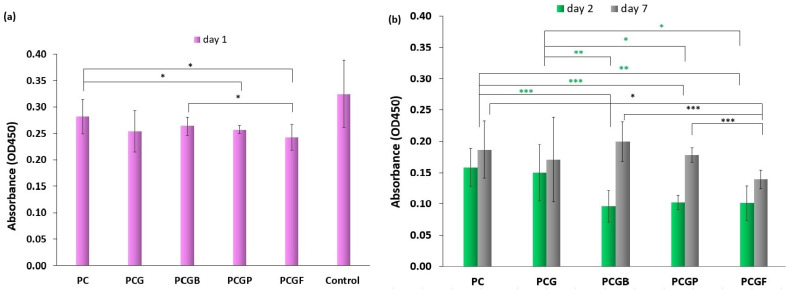
In vitro study of adhesion and proliferation on different composite films. (**a**) adhesion (day 1) and (**b**) short-term and long-term proliferation (day 2 and 7). Significance levels are denoted as * *p* < 0.05, ** *p* < 0.01, and *** *p* < 0.001.

Cell adhesion and proliferation are important factors for the integration of a scaffold into a biological environment [49]. These parameters were evaluated using the CCK-8 assay. The results were analyzed after 24 h (adhesion), 48 h (short-term proliferation) and 7 days (long-term proliferation). The results in Figure 4a revealed the initial cell adhesion of the samples. The adhesion of the porcine and fish gelatin containing samples were lower in comparison to the basic PCL matrix (*p* < 0.05). The PCGF sample with fish gelatin showed an even more reduced adhesion than the PCGB sample containing the bovine gelatin (*p* < 0.05). Based on the previous literature, it was demonstrated that stiffness plays a crucial role in regulating cell growth and leads to higher cell adhesion in the initial step of cell culture [50]. PCL is a hydrophobic synthetic polymer that possesses a surface chemistry capable of absorbing adequate amounts of trace extracellular matrix (ECM) proteins from serum-supplemented media. This property promotes cell attachment [51]. However, when gelatin is incorporated into the PCL matrix, the strength of the composite material decreases. Consequently, the cell adhesion can be decreased.

The short-term proliferation (Figure 4b) of the gelatin composites was lower in comparison to the PCL matrix (PCGB *p* < 0.001, PCGP *p* < 0.001, and PCGF *p* < 0.01). The same trend was seen in the PCG and all the samples with gelatin (PCGB *p* < 0.01, PCGP *p* < 0.05, and PCGF *p* < 0.05).

In the case of long-time proliferation, the highest changes in cell growth were observed in all the samples incorporated with gelatin. The sample with the fish gelatin (PCGF) had a significantly lower proliferation than the mammalian gelatin composites (*p* < 0.001). Considering the overall results, the addition of gelatin, whether bovine, porcine, or fish, had varying effects on cell adhesion. Over time, the addition of the gelatin contributed to an increased cell proliferation when compared to the samples without gelatin. This indicates that gelatin dynamically influences cell behavior and may promote cell proliferation in the long term. These findings suggest that gelatin incorporation promotes cell proliferation and tissue regeneration. Gelatin supports vital biological processes by increasing the surface area for cell binding, leading to an increased cell proliferation and a better potential for successful tissue regeneration [52]. Cell adhesion and proliferation are dynamic processes independent of the current situation of cell growth but are also influenced by the properties of the materials and the conditions in the biological environment during the entire healing process.

### 2.7. Cell Staining

**Figure 5:**
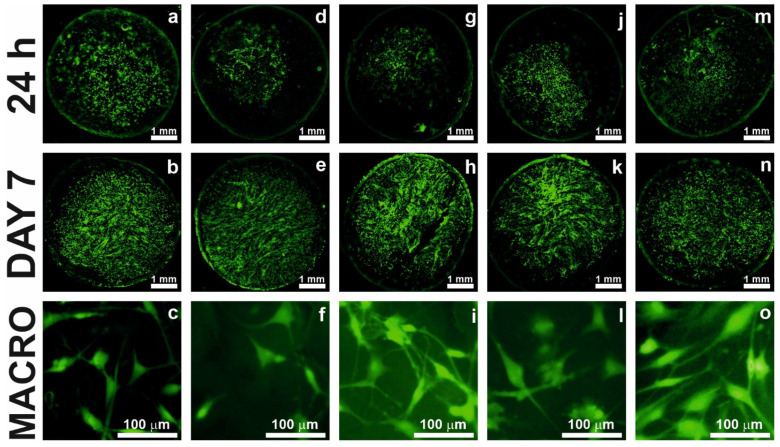
Fluorescence microscopy images of different types of composite films. (**a**–**c**) PC, (**d**–**f**) PCG, (**g**–**i**) PCGB, (**j**–**l**) PCGP, and (**m**–**o**) PCGF. Images in the first and second rows represent cell attachment at 24 h and proliferation on day 7, respectively. The third row provides magnified views of the cells.

Visual comparison of cell attachment, spreading, and proliferation on all the samples was assessed using CellTrackerTM Green fluorescent staining (Figure 5). It was observed that all the samples exhibited the presence of viable cells at 24 h, as indicated by the fluorescent images. Among all, the PC sample (Figure 5a) displayed a relatively higher number of cells, suggesting a stronger initial attachment of cells compared to the other composite films. These findings are consistent with the results obtained from the CCK-8 assay, further confirming the successful attachment and viability of cells on the PC composite film. This observation is in accordance with the previous studies, reporting the biocompatibility and cell adhesion properties of PCL-based materials [53]. The cell proliferation on day 7 significantly increased for all the samples, especially for the sample containing bovine gelatin (PCGB), where it is apparent that the surface of the sample is fully covered with cells throughout (Figure 5h).

The incorporation of gelatin into PCL composite films have been demonstrated to yield improvements in cell performance. These improvements have been supported by various studies, highlighting the excellent biocompatibility of gelatin and its potential for applications in bone tissue engineering [54]. The fluorescence microscopy images provide evidence of the favorable biocompatibility and cell-supporting characteristics of the composite film scaffolds. The cells on the surfaces of all the samples show the correct spindle-like morphology as observed in the last row of Figure 5. These findings are consistent with the previous studies that have highlighted the importance of scaffold bioactivity and biocompatibility in facilitating cell adhesion and tissue regeneration [55].

### 2.8. Cytotoxicity

**Figure 6:**
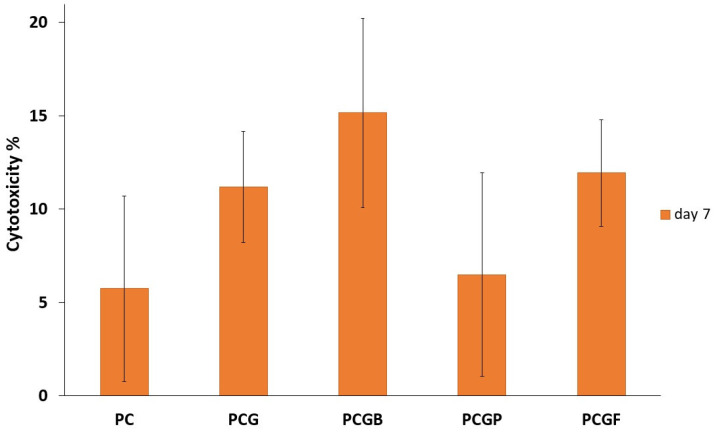
Cytotoxicity of all samples (day 7) expressed as median values of LDH released from HDF cells. The percentage value is between the maximal (100%) and spontaneous (0%) LDH activity.

The LDH activity (Figure 6) of the cells was assessed to compare the cytotoxicity of the various composite films 7 days after seeding. The amount of LDH released is expressed as a cytotoxicity percentage according to Equation (3). Upon reviewing all of the samples, it is evident that each of them exhibits a low level of cytotoxicity. The PC sample exhibited a cytotoxicity of 4.92%, which suggests a very good biocompatibility as described in previous studies [56]. The PCG film showed a slightly higher cytotoxicity of 7.77%, but not significantly different from the basic PCL matrix. Remarkably, the incorporation of gelatin derived from diverse sources resulted in similar level of cytotoxicity compared to the PC and PCG samples (PCGF 3.89%, PCGB 11.07%, and PCGP 5.9%). In general, all the samples exhibited no statistically significant differences in cytotoxicity when compared to each other, which confirmed their similar properties. Fish gelatin possess favorable properties for cultivation of HDFs, resulting in an enhanced biocompatibility. The literature specifically addresses the use of fish gelatin in composite films for bone regeneration, some studies have reported the potential of fish gelatin as a biomaterial with excellent biocompatibility [57].

Moreover, previous studies have reported that the structure of composite films with gelatin is more similar to the proteins in human skin, allowing for an increased compatibility and a decrease in potential side effects. Gel-based scaffolds appear to be a promising material for artificial skin scaffolds, demonstrating a high level of biocompatibility [58].

## 3. Conclusions

In this study, biodegradable composite films were produced by mixing and casting PCL and PLGA with gelatin derived from bovine skin, porcine skin, and fish skin. Despite having good mechanical properties, the films differed in terms of flexibility, stiffness, and toughness. Their tensile strength was suitable for maintaining structural integrity during cell experiments. The thermal stability of the films remained unaffected by gelatin incorporation, ensuring their suitability for various applications. Moreover, the composite films exhibited a desirable biodegradability and high hemocompatibility, with gelatin playing a role in the biodegradability improvement. Importantly, all the composite films promoted cell adhesion, spreading, and proliferation, indicating their potential for tissue regeneration. The results of the cytotoxicity assessment demonstrated that all the composite films fall within the range of safety and viability for tissue regeneration. Overall, this study highlights the potential for gelatin-incorporated composite films as biodegradable and biocompatible materials for tissue engineering applications. Further investigations should focus on elucidating the underlying mechanisms and optimizing the gelatin composition to tailor the film’s properties for specific biomedical applications.

## 4. Materials and Methods

### 4.1. Materials

Polycaprolactone (PCL) (M_w_ = 50,000, powder) and poly (lactic-co-glycolic acid) (PLGA) (70:30, IV 0.2 dL/g) were purchased from Polysciences Europe GmbH (Hirschberg an der Bergstraße, Germany). Gelatin from bovine skin (G9382, gel strength ~225 g Bloom, Type B), gelatin from porcine skin (G2500, gel strength 300, Type A) and gelatin from cold water fish skin (G7041) were obtained from Sigma-Aldrich (Burlington, MA, USA). All chemicals were of analytical grade.

### 4.2. Preparation of Composite Films

The samples were prepared with the compositions as shown in Table 2. The pure PCL polymer was used as the basic sample (PCL: 1 g/7 mL). The PCL and PLGA were dissolved in chloroform (PCL: 0.8 g/5 mL and PLGA: 0.2 g/2 mL). In a separate step, each type of gelatin was dissolved in acetic acid (0.04 g/2 mL). After conducting a series of tests involving different ratios of gelatin in the composite preparation, it was determined that a composition containing 10% gelatin yielded the optimal results based on the homogeneity and scaffold toughness. The mixing process of PCL and PLGA was performed at a temperature of 60 °C with slow stirring by a magnetic stirrer for approximately 30 min to ensure the complete dissolution of the polymers.

Once the PCL and PLGA solutions were mixed together, the temperature was lowered to 40 °C and the gelatin solution was added to the PCL/PLGA mixture. The tri-component solution was stirred for an additional 10 min to achieve a homogeneous distribution of the gelatin in the solution.

**Figure 7:**
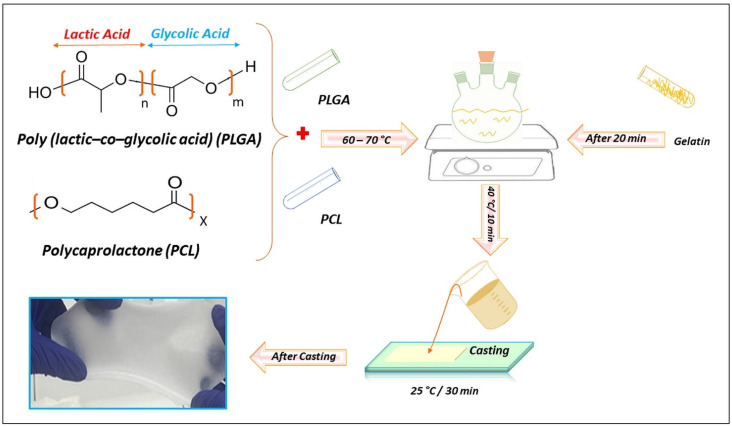
Schematic illustration depicting the fabrication process of composite films consisting of PCL and PLGA with incorporated gelatin.

The resulting solution, which appeared completely homogeneous was promptly poured onto a glass surface of the casting machine AB3120 (Berlin, Germany) followed by drying. The casting machine allowed for the even spreading of the solution, resulting in the formation of films with a 0.5 mm thickness. After pouring the solution onto the casting machine, the films were left undisturbed at room temperature for approximately 30 min. During this time, the films detached themselves from the glass plate of the casting machine, indicating their readiness for further use (Figure 7). The objective was to obtain pure materials and gain a comprehensive understanding of their structure and reaction mechanisms. During the film preparation process, we avoided the use of inorganic nanoparticles, metal binding catalysts, or strong organic chemicals to align with a health-related biomedical approach and prevent potential negative impacts or toxic side effects.

### 4.3. Material Characterization

To examine the surfaces, textures, and depth profiles of the composite films a series of images were taken by a VHX-7000 digital microscope (Keyence Co., Ltd., Osaka, Japan). The imaging was performed in both two-dimensional (2D) and three-dimensional (3D) modes at magnifications of 100x and 300x.

The tensile tests were conducted using a DMA Q800 dynamic mechanical analyzer (TA Instruments, New Castle, DE, USA) at an ambient temperature. The experiment employed square-shaped specimens with dimensions of 6 × 6 mm and a thickness of 3 mm. The gauge length between the clamp jaws was set to 15 mm. A displacement rate of 1 mm/minute was applied during the tests. To ensure accuracy and reliability, each measurement was performed in triplicate, and the average value was recorded.

The thermogravimetric analyses (TGA) were conducted using a thermogravimetric analyzer Q500 (TA Instruments, USA) by heating approximately 5 mg weighing samples from 30 to 800 °C at a ramp rate of 10 °C/min under a nitrogen atmosphere.

### 4.4. Analytic Methods

#### 4.4.1. Biodegradability Test

To assess the biodegradability of the scaffolds, they were soaked in a simulated body fluid (SBF) for 24 days at 37 °C. The SBF was prepared according to the protocol established by Kokubo et al. [59]. For the detailed composition see Table 3.

The samples were dried to a constant weight and immersed into tubes containing 10 mL of SBF, which were then sealed and incubated for 24 days at 37 °C. Subsequently, the scaffolds were dried in a laboratory oven at 40 °C for 1 day to a constant weight. The weight changes were determined using the following Equation (1):

Biodegradability (%) = ((m_i_ − m_f_)/m_i_) × 100 (1)

where $m_{i}$ represents the initial weight and $m_{f}$ represents the final weight of the scaffold after incubation and drying.

#### 4.4.2. Hemolytic Test

The hemolytic test was conducted, according to Chocholata et al. [60]. Briefly, fresh human blood was collected in a tube containing sodium citrate and diluted with a normal saline solution (8 mL blood + 10 mL normal saline). A standard tube containing 10 mL of normal saline was kept in an incubator at 37 °C for 30 min, providing temperature equilibration. To the preheated tube with sample 0.2 mL of the diluted blood was added. The tube was then mixed gently and incubated for 60 min at 37 °C. After incubation, all the test tubes were centrifuged for 5 min at 3000 rpm and 100 μL of the supernatant was carefully removed and transferred to 96-well plate and measured at a wavelength 545 nm on the microplate reader Cytation 5 (BioTek, Winooski, VT, USA) and the hemolysis rate (HR) in % was calculated based on Equation (2):

(2) $$Hemolysis\;Rate\;(\%)=\frac{A_{\mathrm{sample}}-A_{n}}{A_{p}-A_{n}}\times 100$$

where A_sample_ represents the absorbance of the sample, A_n_ refers to the absorbance of the negative control, and A_p_ represents the absorbance of the positive control. As a positive control 0.2 mL of diluted blood was used in 10 mL of deionized water. As a negative control 0.2 mL of diluted blood was used in 10 mL of normal saline solution and incubated for 60 min at 37 °C.

#### 4.4.3. Human Dermal Fibroblasts Culture Conditions

In order to investigate the potential of composites for use in biomedical applications, biological testing was conducted using human dermal fibroblasts (HDFs, Axol Bio, Cambridge, UK). The HDFs were cultured in Dulbecco’s Modified Eagle’s Medium (DMEM, Biosera Europe, France) supplemented with 10% (*v*/*v*) fetal bovine serum, 100 U/mL penicillin, 100 mg/mL streptomycin (all Biosera Europe, Nuaillé, France), and 2 mM stable glutamine (Diagnovum GmbH, Ebsdorfergrund, Germany) under the standard conditions of 37 °C and 5% CO_2_ in a humidified incubator. The culture medium was replaced as necessary.

#### 4.4.4. Cell Adhesion and Proliferation

The samples were sterilized for 60 min, rinsed with deionized water, and placed in a 48-well microplate. Every sample was seeded with HDFs at a density of 3000 cells each. To determine the initial adhesion and proliferation of the seeded cells, a Cell Counting Kit-8 (CCK-8, Bimake, Weinheim, Germany) was used according to the manufacturer’s instructions. For adhesion, after 4 h from seeding, the samples were incubated with a 1 mL CCK-8 working solution at 37 °C. After incubation 110 μL of the solution was transferred to a 96-well plate and absorbance at 450 nm was determined using a Cytation 5 imaging reader. The short-term proliferation of the cells was determined after 48 h and the long-term proliferation 7 days after seeding. The measurement was performed in triplicates.

#### 4.4.5. Cell Staining

To visualize the population density, cultured cells on scaffolds were stained with 4 μM CellTrackerTM Green CMFDA Dye (ThermoFisher Scientific, Eugene, OR, USA) according to the manufacturer’s instructions 24 h, and 7 days after cell seeding. The living cells were observed and photographed using a Leica M205 FCA fluorescent stereomicroscope (Leica Microsystems, Wetzlar, Germany). 

#### 4.4.6. Cytotoxicity Test

For the estimation of cytotoxicity of the samples a CyQUANT LDH Cytotoxicity Assay Kit (Thermo Fisher Scientific, MS, USA) was used. Seven days after seeding, 50 μL of supernatant was transferred to a 96-well plate and 50 μL of the reaction mix was added. After 30 min of incubation, a stop solution was added and an absorbance at 490 nm and 680 nm was measured immediately. For the estimation of spontaneous LDH activity, cells on 48-well plate were treated with deionized water (in the ratio 1:10 to medium). For the estimation of maximal LDH activity, cells on a 48-well plate were treated with a lysis buffer (in the ratio 1:10 to medium). To determine the LDH activity, the absorbance at a wavelength of 680 nm was subtracted from the absorbance of 490 nm. Cytotoxicity in % was calculated according to the following Equation (3):

(3) $$Cytotoxicity\;\%=\frac{A_{\mathrm{sample}}-A_{spontaneous}}{A_{max}-A_{spontaneous}}\times 100$$

where A_sample_ is the absorbance of the sample, A_spontaneous_ is the absorbance of the negative control, and A_max_ is the absorbance of the positive control.

## Figures and Tables

**Table 1:** Results of biodegradability assessment and the test of hemocompatibility.

Samples	Composition Materials (PCL/PLGA/Gelatin Type)	Biodegradability [%]	Hemolysis * [%]
PC	(PCL/PLGA/-)	11.70 ± 0.07	4.6 ± 0.2 (+++)
PCG	(PCL/PLGA/-)	15.15 ± 0.08	4.3 ± 0.3 (+++)
PCGB	(PCL/PLGA/Bovine)	16.22 ± 0.07	4.7± 0.5 (+++)
PCGP	(PCL/PLGA/Porcine)	21.50 ± 0.09	4.3 ± 0.2 (+++)
PCGF	(PCL/PLGA/ Fish)	28.40 ± 0.10	4.2 ± 0.3(+++)

* Highly hemocompatible (<5% hemolysis, +++), hemocompatible (within 10% hemolysis, ++), and nonhemocompatible (>20% hemolysis, +). Values represent the mean ± SD, n = 3.

**Table 2:** Composition of the prepared composite films.

Samples	PCL [wt.%]	PLGA [wt.%]	Bovine Gelatin [wt.%]	Porcine Gelatin [wt.%]	Fish Gelatin [wt.%]
PC	100	-	-	-	-
PCG	80	20	-	-	-
PCGB	80	10	10	-	-
PCGP	80	10	-	10	
PCGF	80	10	-	-	10

**Table 3:** Composition of SBF to make 500 mL solution.

Order	Reagent	Amount
1	NaCl	4.018 g
2	NaHCO_3_	0.178 g
3	KCl	0.114 g
4	K_2_HPO_4_ 3H_2_O	0.116 g
5	MgCl_2_ 6H_2_O	0.159 g
6	1M HCl	19.5 mL
7	CaCl_2_	0.146 g
8	Na_2_SO_4_	0.036 g
9	Tris	3.059 g
10	1M HCl	1.84 mL

## Data Availability

The data presented in this study are openly available in article.
